# Implementation of WHO guidelines for cervical cancer screening, diagnosis and treatment: knowledge and perceptions of health providers from Argentina

**DOI:** 10.1186/s12885-024-12650-7

**Published:** 2024-08-12

**Authors:** Silvina Arrossi, Cecilia Straw, Victoria Sanchez Antelo, Melisa Paolino, Armando Baena, Mathilde Forestier, Maryluz Rol, Maribel Almonte

**Affiliations:** 1 Centro de Estudios de Estado y Sociedad/Consejo Nacional de Investigaciones Científicas y Técnicas, Buenos Aires, Argentina; 2 Centro de Estudios de Estado y Sociedad, Buenos Aires, Argentina; 3 International Agency for Research on Cancer, Lyon, France; 4 Department of Sexual and Reproductive Health and Research, World Health Organization, Geneva, Switzerland

**Keywords:** WHO cervical cancer guidelines, Implementation, Dissemination, Adoption, Argentina

## Abstract

**Background:**

The 2021 World Health Organization (WHO) guidelines on cervical cancer screening and treatment provide countries with evidence-based recommendations to accelerate disease elimination. However, evidence shows that health providers’ adherence to screening guidelines is low. We conducted a study in Argentina to analyze health providers’ knowledge and perceptions regarding the 2021 WHO Guidelines.

**Methods:**

A qualitative study was conducted based on individual, semi-structured interviews with health providers specializing in gynecology (*n* = 15). The themes explored were selected and analyzed using domains and constructs of the Consolidated Framework for Implementation Research.

**Results:**

Although health providers perceive WHO as a reliable institution, they do not know the 2021 guidelines, its supporting evidence, and its elaboration process. Their clinical practice is mainly guided by local recommendations developed by national professional medical associations (PMAs). For interviewees, WHO guidelines should be disseminated through health authorities and national PMAs, mainly through in-service training. Health providers had a positive assessment regarding WHO Recommendation 1 (screen, triage, and treatment for women aged 30 + with HPV-testing every 5 to 10 years) and perceived a favorable climate for its implementation. HPV-testing followed by triage was considered a low-complexity practice, enabling a better detection of HPV, a better selection of the patients who will need diagnosis and treatment, and a more efficient use of health system resources. However, they suggested adapting this recommendation by removing screening interval beyond 5 years. WHO Recommendation 2 (screen-and-treat approach with HPV-testing for women aged 30 + every 5 to 10 years) was predominantly rejected by interviewees, was considered an algorithm that did not respond to women’s needs, and was not adequate for the Argentinean context. Regarding the HPV-test modality, clinician-collected tests were the preferred mode. Health providers considered that HPV self-collection should be used primarily among socially vulnerable women to increase screening coverage.

**Conclusion:**

WHO guidelines should be widely disseminated among health providers, especially in settings that could benefit from a screen-and-treat approach. Identifying areas of partnership and collaboration with PMAs in implementing WHO guidelines is essential.

**Supplementary Information:**

The online version contains supplementary material available at 10.1186/s12885-024-12650-7.

## Background

Cervical cancer is a preventable disease, and its elimination depends on global and local political will to drive transformative actions aimed at improving the quality of healthcare and contributing to reducing health inequities [1]. The utilization of guidelines has the potential to diminish inappropriate practices, minimize unwarranted practice variation, enhance the translation of research into practice, and improve healthcare quality and safety [2].

The World Health Organization (WHO) guidelines offer evidence-based recommendations for clinical practice and/or public health policies. These recommendations inform clinicians and policymakers about the decisions they can or should make in specific situations to achieve optimal health outcomes [3]. The guidelines present choices among various interventions or measures expected to impact health outcomes and resource utilization positively. In May 2020, WHO launched the Global strategy to accelerate the elimination of cervical cancer (CC), including targets for each of the three pillars for 2030: 90% human papillomavirus (HPV) vaccination coverage of eligible girls, 70% screening coverage with a high-performance test and 90% of women with a positive screening test or cervical cancer cases managed appropriately.

One key activity of the CC elimination strategy was to update the 2013 WHO guidelines for screening and treatment to prevent CC, and to simplify the algorithms [4, 5]. Thus, in 2021, the revised version of the WHO guidelines was published, with HPV-testing recommended as a main strategy for screening, to be implemented as part of a screen, triage and treat strategy or as part of a screen-and-treat strategy according to local needs, capacities, and resources of different settings [5]. These guidelines target a broad range of stakeholders responsible for choosing strategies for CC prevention at the country, regional, and district levels [6].

Despite the importance of guidelines to ensure the provision of effective screening practices that reach populations at the highest risk, evidence shows that, in general, health providers (HPs) have low adherence to CC screening guidelines [7, 8]. Factors explaining this low adherence include insufficient awareness of the current scientific evidence, limited access to updated guidelines, and low commitment toward ongoing education requirements [9, 10]. This low adherence coexists with the development of guidelines by national, regional, and international institutions that, although most are aligned with each other, sometimes present discrepancies [11–13]. A systematic review identified differences across national screening guidelines from 11 countries in North America, Europe, and the Asian-Pacific region. The main discrepancies were observed in screening start and end age, intervals, and primary screening methods [12]. These variations among countries, jurisdictions, and even within different healthcare services have been identified as inefficient in the utilization of healthcare services [14], leading to the perpetuation and exacerbation of health inequities [15].

Improving adherence to guidelines entails empowering clinicians to make decisions based on the most effective and up-to-date evidence and dissemination of guidelines [14]. At the institutional and system levels, guidelines’ dissemination involves establishing a context that supports their implementation and a political consensus to translate the recommendations into scalable health policies [9, 16]. Therefore, incorporating WHO guidelines as a routine programmatic public health policy will depend on guidelines dissemination by national and international agencies, stakeholders’ knowledge and perceptions about its recommendations, as well as barriers and facilitators of their implementation. Evaluating stakeholders’ perceptions of the 2021 WHO guidelines is key to ensuring a high level of adoption, especially as they include recommendations targeted at settings with different levels of resources. However, very little knowledge exists about the adoption of WHO CC screening guidelines.

In this paper, we present the results of a study conducted in Argentina aimed to analyze knowledge and perceptions regarding the 2021 WHO Guidelines by HPs, as well as obstacles and facilitators for its adoption.

## Methods

### Theoretical perspective

In this paper, we present results from the semi-structured interviews with HPs carried out to gather information about their perceptions regarding the implementation of WHO guidelines. The themes explored were selected and analyzed using the domains and constructs of the Consolidated Framework for Implementation Research (CFIR) [17], which offers analytical tools appropriated to understand contextual factors working for or against implementation efforts. It is a ‘meta-theoretical’ framework that provides a set of constructs arranged across five domains: (1) those related to the *intervention characteristics*; (2) the *outer setting* that is comprised of the social, political, and economic situation of the organization in which the intervention will be implemented; (3) the *inner setting* includes the political, cultural and structural atmosphere through which the intervention will be processed; (4) the *characteristics of the individuals* involved in the implementation of the intervention; and (5) the *process* for the implementation and executing the plan as it was designed are constructs within the process domain. From each domain, we selected the constructs that were most relevant to our study (Table 1) [18].

**Table 1:** CFIR domains and construct definitions used for data collection

Domains and Constructs		Definitions used
**I. Intervention characteristics**		
A	Intervention Source	Trust in WHO as the guideline source (legitimacy, familiarity with the local context).
B	Evidence Strength & Quality	Trust in the evidence that supports preferred/OMS guidelines.
C	Relative Advantage	The advantage of implementing WHO Recommendations 1 and 2 versus an alternative solution and/or current usual practices.
D	Adaptability	Perception about the degree of adaptability of 2021 WHO Recommendations 1 and 2.
F	Complexity	Perception about practicability and usability of the 2021 WHO Recommendations 1 and 2: how complex they are to use.
G	Design Quality & Packaging	Perception about the format and how easy is to access the 2021 WHO Guidelines.
H	Cost	Perception about that implementation of WHO Recommendations 1 and 2 as requiring additional resources
**II. Outer setting**		
A	Patient Needs & Resources	Perception about how WHO Recommendations 1 and 2 apply to the local setting. Perception about how 2021 WHO Recommendations 1 and 2 fit patient needs.
D	External Policy & Incentives	Policies and incentives needed for implementation of Recommendations 1 and 2. Existence of national/provincial policies, norms, and regulations regarding practices.
**III. Inner setting**		
D	Implementation Climate	Shared receptivity by the organization management/health authorities regarding the use of guidelines in general, and the 2021 WHO Guidelines in particular.
E3	Access to Knowledge & Information	Perceptions about channels to access the 2021 WHO Guidelines.
**IV. Individuals characteristics**		
A	Knowledge & Beliefs about the Intervention	Provider knowledge, beliefs and attitudes regarding the recommendations included in the 2021 WHO Guidelines.
B	Self-efficacy	Provider belief in their own capabilities to implement WHO Recommendations 1 and 2.

*Source* Adapted from Damschroder et al. [18]

### Setting

Argentina is a federal country made up of 24 provinces, each of which is an autonomous entity responsible for the organization, management, and financing of the provincial health system. The National Ministry of Health provides a regulatory framework for healthcare provision, as well as training and financing for specific programs through nationally and internationally funded initiatives. Provincial health ministries can choose whether to adhere to the proposed national health programs or activities; their adherence mainly depends on formal agreements in which responsibilities and funding are negotiated.

Since 2012, National authorities have established HPV-testing as the primary CC screening test for women aged 30 years or older attending public health centers. Women are screened with HPV-testing every five years. According to Argentinean recommendations [19], HPV-positive women are triaged with cytology. Women with abnormal Pap smears are referred to colposcopy and biopsy if needed. When lesions are histologically confirmed as cervical intraepithelial neoplasia 2 or worse, women are referred for treatment. HPV-negative women are recommended to be re-screened in 5 years. HPV-positive women with normal cytology are recommended to be re-screened in 18 months. All HPV-testing, diagnoses, and treatments of women screened in the public health system are registered on the national screening information system (SITAM, for its initials in Spanish). Results of HPV tests and triage Pap smears are instantly available online to health providers at public health establishments. At present, 19 out of 24 provinces offer HPV-testing in public health services.

The study took place in the Metropolitan Area of Buenos Aires (AMBA), the largest metropolitan region in Argentina, with a population of over 14 million people. AMBA encompasses the Autonomous City of Buenos Aires (CABA, for its initials in Spanish) and 24 districts in the Buenos Aires province (Great Buenos Aires, GBA, for its initials in Spanish). In six districts of the AMBA, HPV testing was implemented as the primary screening.

### Participants

We conducted 15 online semi-structured interviews with health providers who specialize in gynecology. The selection of participants followed a purposive sampling approach, considering their relevance and theoretical saturation regarding CFIR constructs. We aimed to ensure a diverse range of responses and facilitate meaningful comparisons among the participants.

### Data collection

**Fig. 1:**
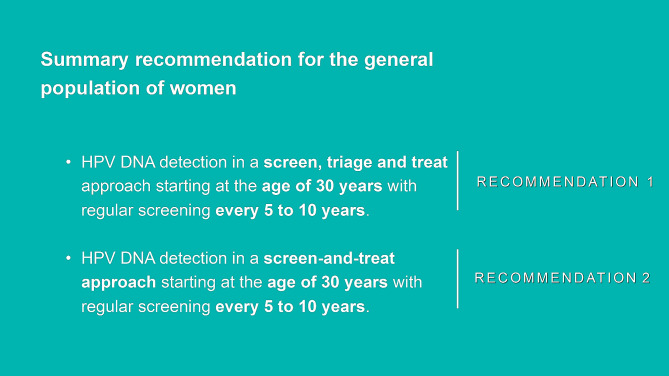
Summary recommendations for screening and treatment approaches. *Source* Adapted from 2021 WHO Guidelines [5]

The interview guide was organized based on the CFIR constructs (Table 1). We employed the CFIR construct for: (1) evaluating knowledge and attitudes regarding the 2021 WHO guidelines; (2) analyzing perceptions about the summary recommendations for the general population provided in the guidelines (Fig. 1). (3) identifying possible channels and formats for disseminating the 2021 WHO guidelines. Two pilot interviews were conducted, based on which adjustments were made to the interview guide.

Fieldwork was carried out between June and August 2022 by a female social science researcher. She invited participants through e-mail/WhatsApp to participate in an online interview through a virtual platform (Zoom). Interviews lasted on average one hour; permission was obtained from participants to audio-record them.

### Data analysis

The collected data were analyzed thematically. Once the interview transcriptions were completed, all interviews were entered into the software. The coding and analysis followed the domains, constructs, and dimensions of CFIR.

Interview audios were transcribed verbatim to carry out thematic analysis of the debates [20], based on an iterative and flexible process following six steps: (1) To ensure coding reliability, two researchers (authors CS and VSA) become independently familiar with the data through transcriptions and the video recording. (2) We classified data using an initial codebook based on the CFIR constructs (Table 1), and following our research objectives, we identified the professionals’ opinions. (3) We analyzed each category to generate new themes understated as “a salient aspect of the data in a patterned way, regardless of whether that theme captures the majority experience of the participants” [20]. (4) Both researchers met to review themes to identify consistencies and resolve the inconsistencies with the other team members (MP and SA). (5) We grouped the emergent themes according to their conceptual similarities to define and name the subthemes. (6) We sought examples that adequately graphed each theme. Several group meetings were held to discuss the results and prepare the final manuscript.

We used ATLAS.Ti (version 7.5.4; Scientific Software Development GmbH, Berlin) to organize, code, and summarize patterns. To ensure coding reliability, a third author (SA) verified coding against a sample of transcripts and critically reviewed the data and themes to improve study trustworthiness [21]. The details of the method and results are presented following the Consolidated Criteria for Reporting Qualitative Research (COREQ) [22].

The study’s protocol was approved by the Gino Germani Institute’s ethics committee from the Faculty of Social Sciences, University of Buenos Aires. Participants provided written informed consent. The anonymity of participants was guaranteed at each step of the study.

## Results

### Participant’s characteristics

Fifteen health providers working in the public health system were interviewed, 11 women and four men; the mean age was 47.6 years (range 33 to 63 years). The average amount of time that they had occupied their roles was 11 years. Six HPs used HPV-testing as primary screening.

### Context and preferences for the application guidelines to prevent CC

When asked about the guidelines they use to decide their clinical practice, the interviewees mentioned a decision-making process based on their training and local guidelines, preferably those elaborated by national professional medical associations (PMAs). For them, these are: (1) recommendations developed by entities with scientific prestige and impact on both training and clinical practice; (2) they are suitable for the characteristics of the patients (number of sexual partners, infrequent users, low educational level, social and economic vulnerability), and the local socio-economic context; and (3) they provide legal backing for clinical practices. Some HPs also mentioned using guidelines developed by national and provincial health authorities.

“… I try to base myself on what the guidelines, protocols, the National [Ministry of Health] guide, professional consensus, updates, what the Argentinean Federation of Gynecology and Obstetrics Societies state, a little bit of everything…” [Professional 2, CABA]

### Perceptions of the WHO guidelines

#### Knowledge, quality, and strength of supporting evidence, peer pressure, and origin of intervention

In general, the WHO was perceived as a reliable scientific institution for the development of screening and treatment guidelines to prevent CC, although interviewees were not familiar with the development process, nor was the evidence supporting its recommendations. In this regard, most of them were unaware of the WHO 2013 and 2021 guidelines and experiences of concrete use. In contrast, there was greater knowledge of the WHO guidelines on other pathologies (e.g., contraception). The interviewees considered that, although it is positive to have global guidelines for the prevention of CC, international recommendations such as those of the WHO are very general and difficult to apply in local realities. National guidelines are perceived as more applicable and adapted to clinical practice.

“I always considered the WHO as a reliable institution” [Professional 6, GBA]

“I don’t know the WHO guidelines” [Professional 12, GBA]

“The truth is that I have no idea [of experiences of using the WHO guidelines for cervical cancer]” [Professional 10, GBA]

Those isolated HPs who were familiar with the WHO guidelines, rated their development process, evidence-based content, and design quality very positively. In these cases, access to the guidelines was eased by the dissemination carried out by the National Cancer Institute during the implementation of the HPV Test.

“… the guidelines are very good, the scientific evidence and the rationale for developing them.” [Professional 1, CABA]

### Perceptions on recommendation 1: screen, triage and treat approach for women aged 30 + every 5 to 10 years

#### Relative advantage and patients’ needs

For interviewees, the main advantages of this strategy are that it enables: A better detection of HPV by using a highly sensitive test; a better selection of the patients who will need to continue in the line of care, and the possibility of earlier diagnoses; the extension of the screening interval to five years, compared to the annual Pap of the current practice; a more efficient use of health system resources, by saving resources allocated today to more frequent screening, which could be assigned to expand screening coverage; to select patients according to the oncological risk, and to treat only those patients who need it.

“Knowing that with the HPV test, they don’t have to come back as often and can come back in five years” [Professional 13, GBA]

“It would favor HPV detection a lot because cytology and colposcopy are very operator-dependent. The HPV test is quite reliable and trustworthy” [Professional 4, GBA]

“It would allow to have more human resources and more supplies to see more patients, and expand coverage” [Professional 1, CABA]

These relative advantages would result in greater preventive control of the population, and therefore they consider the recommendation to be responsive to women’s needs.

Regarding the age of screening included in the recommendation, some interviewees considered 30 years to be an advantage because infections in younger women are transient and can be resolved without interventions. Others considered screening might be too late at that age, which was seen as a disadvantage.

“We have had cervical cancer in very young patients aged 25, 24, 27, 32. Perhaps because of the early onset of sexual relations, because of low immunity due to a poor diet, stress, a loss of vitality or living conditions, there have been many cases [of cancer]” [Professional 6, GBA]

In relation to the screening interval, the HPV test every 5 years was found to be a reliable and safe period. On the other hand, the 10-year interval was unanimously rejected, as it would not guarantee preventive care for women.

“[…] 10 years is not so reliable, you have to take into account sexual activity, the change of partners, the possibility that the test is not negative and is positive” [Professional 4, GBA]

#### Adaptability

In line with the perceived disadvantages of screening every 10 years, there was a unanimous suggestion to adapt Recommendation 1 by removing the possibility that screening could be extended beyond five years. Furthermore, another adaptation mentioned was not to set 30 years as the starting age for screening, but to be able to decide after assessing the patient’s characteristics (e.g., age of first intercourse, number of sexual partners, etc.).

“I would include some characteristics of the patient: age of onset of sexual relations, multiple partners… to know if it is worth doing it before the age of 30” [Professional 5, GBA]

#### Complexity and self-efficacy

There was consensus among respondents that performing an HPV test would have low or no complexity because of its similarity to performing a Pap test. They considered that a gynecologist has the necessary skills to perform the HPV test, without requiring additional training. Triage and treatment of histologically confirmed patients would also be of no complexity as these practices are in line with the current algorithm for the CC approach. However, some interviewees highlighted the complexity of the changes needed to introduce it into the health system in terms of logistics and administrative management, clinical practice, and the installation and reorganization of laboratories. Other interviewees also highlighted the complexity of de-implementing the annual Pap/colposcopy, as it is an established preventive practice in the population as well as among HPs. In this sense, training would be necessary to incorporate the new aspects and scientific foundations of the HPV test.

“This implies stopping doing Pap smears and colposcopy together, an important change in management, the cytology services have to be adapted, the laboratories have to incorporate all this. We are only going to get that 12% of [HPV+] women [for diagnosis]” [Professional 3, CABA]

#### Costs

For the majority, the implementation of Recommendation 1 requires additional resources including the HPV Test, human resources (e.g., to transport and process samples, and upload screening information), and financial resources to equip/install laboratories, train health teams, adapt doctor-patient communication, and conduct information and promotion campaigns to the community.

“Obviously you would need more resources to rearrange the whole system, the laboratories, a lot of training and awareness-raising for health personnel to be able to implement it… Screening alone is not enough, it has to be accompanied by other things…” [Professional 3, CABA]

### Perceptions of recommendation 2: screen-and-treat approach for women aged 30 + every 5 to 10 years

#### Relative advantage and patients’ needs

For most respondents, this recommendation would have no advantages over the currently used strategy and does not respond to women’s needs. There was a predominance of complete rejection as HPs considered it an inadequate algorithm. Without triage, they would not be able to diagnose the patient and define the treatment. This would imply overtreatment of patients, who may have no lesions or have transient lesions that could be resolved by the action of the immune system and without any treatment. Unnecessary treatment could cause reproductive difficulties for women and thus become an unsafe clinical practice. On the other hand, for them, the evidence does not show that an earlier patient intervention changes the disease’s future evolution, nor does the time of the natural history of the disease justify the urgency of treatment. Moreover, they also mentioned that treating all HPV + women would imply an increase in the number of treatments, which would force the health system to allocate more financial resources to provide an adequate response. However, despite the widespread perception that Recommendation 2 would not have advantages, some isolated HPs mentioned that it might be appropriate for health systems with very limited resources, as it would facilitate the treatment of patients with barriers to accessing health services.

“It has not been proven that treating a low-grade lesion changes the pace of the disease, and it takes many years of untreated HPV lesions to develop into cervical cancer” [Professional 4, GBA]

“Treating the patient just because they have the HPV + test leads to a lot of unnecessary treatment and can affect morbidity and difficulties in getting pregnant. We only treat women who we confirm have something. This is the most accurate way to ensure the safety of the patient and not to hurt her” [Professional 3, CABA]

“Maybe in some kind of population with very difficult access to health centers” [Professional 12, GBA]

“[The WHO] drastically changes the paradigm, clearly seeks to expand coverage, and the risk-benefit balance between treating and not treating. They show with evidence that this type of management [Screen and treat] is efficient” [Professional 1, CABA]

#### Adaptability

The respondents did not consider Recommendation 2 to have adaptable components. For them, the non-triage modality does not align with how clinical practices are organized and resourced, and the increase in treatments would lead to work overload at the second level of care.

#### Complexity and self-efficacy

Most interviewees did not consider it appropriate to assess the complexity of a strategy that they rejected outright. Those who believed it appropriate mentioned that it would be very difficult to implement, given the complex reorganization of current screening, triage, diagnosis, and treatment. In terms of perceived self-efficacy, they noted that they would need additional training to treat positive women, as they are currently referred to the second level of care.

#### Costs

Respondents agreed that implementing Recommendation 2 would require additional financial resources to cover extra time for professionals to perform treatments and acquire the necessary instruments and equipment.

#### External policies and incentives, climate for implementation of the WHO 2021 recommendations

Interviewees agreed that the main external policy needed for the implementation of Recommendation 1 is for the National Ministry of Health to establish HPV-testing as a public policy, as well as a regulation describing its use in clinical practice. They further described that the authorities already recognize the HPV Test as a cost-effective screening practice based on evidence and with consensus for its implementation. On the other hand, they considered that its introduction would be accompanied by a protocolization of current clinical practices, an aspect positively valued by authorities and health teams. In this sense, all interviewees perceived a favorable climate for its implementation. Only one HP mentioned having the perception of resistance to change on the part of the authorities of the health institution where she carries out her clinical practice, which is linked to problems related to the administration of that institution.

“There has to be regulation, and it has to be enforced. Always from the national Ministry from to bottom. It goes to the province and then to the municipality” [Professional 5, GBA]

Although there was a prevailing lack of knowledge about PAHO’s actions to implement the HPV test in the region, the interviewees pointed out that PAHO could play an important role in disseminating the contents and training health teams. They also suggested that PAHO could fund research to produce evidence on effective strategies to implement the guidelines and introduce HPV-testing as primary screening.

On the other hand, it was not possible to assess the climate of implementation and external policies regarding Recommendation 2, as it was rejected by the interviewees.

### Preferences about the modality of HPV-testing (clinician-collected vs. self-collection)

Clinician-collected test collection was the preferred mode. Respondents felt that HPV self-collection should be used primarily among socially vulnerable women to increase screening coverage.

“I don’t have a problem with the patient taking the test herself, it lowers the sensitivity a little bit, but not significantly. And it is a useful study and another tool to increase coverage in cases where access to the system is difficult” [Professional 1, CABA]

“It seems to me that if a patient does not come to the hospital, I would rather have her do a self-collected test than not have any control at all” [Professional 11, GBA]

### Proposed means of dissemination and format of the WHO guidelines

For the HPs, the dissemination of the guidelines should be done by health authorities and national PMAs, mainly through training of health services.

“The professional medical associations and the Ministry of Health of the Nation or the City of Buenos Aires are the best way to disseminate the guidelines and provide professionals with more information and fundamentals” [Professional 1, CABA]

Concerning format and design, they prefer a digital format via the Internet. For some professionals, it could be useful for the contents of the guidelines to be made available on an app, while for others, having the algorithms printed on posters in the consulting rooms is the easiest way to access the contents of the guidelines.

## Discussion

In 2021 WHO updated its 2013 guidelines on CC screening and treatment to provide countries with evidence-based recommendations on the most effective strategies to accelerate disease elimination [5]. However, the translation of these guidelines into practices provided by HPs during health service provision will be highly influenced by their perception and acceptability. To our knowledge, this is the first analysis of HP’s perceptions regarding WHO guidelines for screening and treatment of cervical cancer. For this, we used the CFIR [18], a conceptual framework from implementation science appropriate for evaluating the implementation of health interventions. Results showed that although HPs perceive WHO as a reliable institution, they do not know the CC screening and treatment guidelines, their supporting evidence, and elaboration process. Their clinical practice is guided by local recommendations, mainly those developed by national PMAs. Regarding summary recommendations included in the 2021 WHO guidelines, in general, HPs accept using HPV-testing every 5 years for women aged 30 and over, and are against the possibility of extending the screening interval to ten years. Although some HPs recognized that eliminating triage and treating all HPV-positive women might be useful in low-resource settings, most reject the possibility of implementing this strategy in the Argentinian context. In the country, colposcopy and histology are widely available, especially in the AMBA region where the study was carried out. WHO should make efforts to disseminate evidence regarding the screen-and-treat approach which may be more feasible and cost-effective in areas with limited diagnosis services.

In our sample of HPs, almost no one knew WHO guidelines. These are intended primarily for stakeholders responsible for choosing strategies for cervical cancer prevention, at country, regional, and district levels. Thus, historically, WHO work to disseminate and implement the guidelines has targeted primarily national health authorities. HPs’ lack of knowledge can be attributed to the fact that they have not been directly targeted by WHO dissemination activities. However, Greenhalgh et al. [23] have pointed out that before knowledge can contribute to change initiatives, it must enter the stock of knowledge constructed and shared by the individuals. In addition, the competence of individuals to judge the effectiveness of an intervention is facilitated by their understanding of underlying principles that justify using the intervention. The study also found that despite lacking knowledge regarding CC WHO guidelines, HPs rely on WHO as a scientific institution. The evidence shows that the legitimacy of the origin of an intervention is associated with success in its implementation [24]. Therefore, dissemination among HPs of WHO screening guidelines, its supporting evidence and elaboration methodology might facilitate and accelerate the incorporation of recommendations. However, the challenge for innovations proposed by external entities is the articulation with local stakeholders for dissemination and their involvement in decision-making during implementation [25]. According to Helfrich et al., [26] the lack of involvement of adopters in dissemination and implementation greatly undermines the legitimacy of the external source proposing the innovation. A study that analyzed stakeholders’ acceptability of a mobile health intervention to increase adherence to triage among HPV-positive women found that the intervention being proposed by prestigious external institutions and then designed and implemented collaboratively through consensus with local institutions and health providers was perceived as a facilitator [27]. In our study, respondents also pointed out they perceived WHO recommendations as too general and not for specific local contexts. When knowledge can be codified and transferred across contexts, implementation is more likely to be successful [23]. Hence, dissemination of WHO guidelines might include examples of how they apply to the health and resource situation of specific countries, or regions within countries; and its implementation should include involvement of different local stakeholders and adopters.

In Argentina, the recommendation by the Argentinean National Cancer Institute agrees with the WHO Summary Recommendation 1 to introduce HPV-testing for women aged 30 years and over every 5 years, and this agreement is probably the basis for its high acceptance in our sample of HPs. For them, this strategy offers important relative advantages in relation to the current situation and fits women’s needs, with improved control and increased coverage. These findings are important because stakeholder’s acknowledgment of the relative advantages of an intervention constitutes a sine qua non for its implementation/adoption [23]. However, interviewees did not accept extending the interval to ten years. A 10-year interval is much longer than the annual cytology-based screening interval that is still the predominant usual practice in the country, and twice longer than the interval recommended for HPV-testing by the Argentinean Ministry of Health (every 5 years). In a context where interviewees do not know the scientific basis and the elaboration process of WHO guidelines, they feel their women would not be appropriately cared for with such a long screening interval. This is also the basis for some HPs’ reluctance to begin HPV-testing at 30 years and not to screen women at younger ages. Similarly, a study that analyzed attitudes, practice patterns, and barriers related to cervical cancer screening guidelines among U.S. obstetrician-gynecologists found that physicians were concerned that patients would not receive adequate screening if intervals were extended [28]. Another study that examined adherence to mammographic recommendations in the US found that fear of missing cancer diagnoses and malpractice were behind screening practices that differed from the age and intervals recommended in guidelines [29]. In their analysis of factors influencing adherence to antibiotic prescription guidelines, Wood et al. [30] pointed out that strategies to increase guidelines utilization should train HPs so they feel empowered and have confidence that they are implementing best practices in line with international standards while at the same time maintaining the safety of their patients. For Wood et al., [30] this requires awareness raising of the consequences resulting from prescription outside of guideline recommendations, knowledge building of the recommendations set out in guidelines, and training provision on how they can be adapted to the context.

Evidence has also shown that access to knowledge is more effectively related to the implementation of interventions when combined with leadership engagement [18, 31]. In Argentina, HPV-testing was introduced in 2011–2014 through the Jujuy Demonstration Project led by the National Program on Cervical Cancer Prevention [32]. International evidence on HPV testing performance was the backbone of the project [33]. It was the basis for the national regulation to introduce HPV testing and was disseminated among health authorities and providers through several scientific meetings, seminars, and workshops with the participation of well-renowned national and international scientists, and international agencies such as IARC/WHO and the Pan American Health Organization [33, 34]. This leading role of national authorities, with active dissemination of research evidence with international institutions playing important roles in sensitizing policymakers and supporting the policy process has been identified as a main factor for HPV-testing acceptability, and it is probably the basis of the high acceptance of Recommendation 1. This is coincident with results from a study that showed that proactive national leadership engagement with the involvement of UNICEF and PAHO in presenting global evidence and new WHO recommendations were key drivers behind the implementation of WHO recommendations for childhood pneumonia and possible serious bacterial infection in Bangladesh [31].

Regarding Recommendation 2, for the interviewees, there was not enough scientific evidence to support the implementation of the screen-and-treat strategy. In Argentina, there has not been wide knowledge dissemination and leadership engagement for the screen-and-treat modality included in Recommendation 2. This might partially explain HP’s rejection found in the study, even though the strategy could facilitate CC prevention in country areas that lack human resources, equipment, and instrumental to perform colposcopies and biopsies. A basis for this non-acceptance can also be related to the perception of the screen-and-treat strategy as not fitting women’s needs, as for interviewees it may result in overtreatment and harm to the reproductive health of their patients. In the screen-and-treat approach, WHO recommends the use of ablative treatment when possible. Insufficient knowledge of HPs about the reduced impact of ablative treatments on reproductive morbidity in a subsequent pregnancy [35] might also explain their negative reaction to the strategy.

The complexity of an intervention and its compatibility with existing practices are concepts related to factors that can promote or hinder implementation [36]. Evidence has shown that their use is facilitated when there is a good perceived fit between e-health systems and workflows, and when systems positively influence workplace efficiency [37]. Our results showed that contrary to perceptions regarding WHO Recommendation 2, WHO Recommendation 1 was perceived by HPs as highly compatible with health services organization and ways of functioning. They also considered that Recommendation 1 might be easily integrated into regular health service processes. Complexity is also increased by the process length, i.e., the number of sequential subprocesses and actors involved in health service provision [38]. Some interviewees signaled this process’s complexity by highlighting that HPV-testing complexity lies in changes needed for its successful intervention, e.g., changes in logistic and administrative procedures, laboratory reorganization, etc. Thus, our results suggest that, for successful implementation of HPV-testing, there should also be modifications at the health system level that must accompany its introduction. Also, countries wishing to use WHO Recommendation 2 should make additional efforts to increase HPs’ awareness of their advantages in some specific areas and what it entails regarding health service reorganization.

Our study showed that HPs accepted WHO recommendation regarding HPV-self collection tests, which they see as a tool to increase access to screening among socially vulnerable women. Similar results were shown by a study that analyzed stakeholders’ acceptability of HPV-testing in Argentina, which showed that stakeholders saw self-collection as a tool that gave the health system the possibility to reach a population that is usually out of reach and, in this way, produce a real change in the burden of the disease [33] However, some interviewees also pointed out that HPV-self collection should be used only in cases where clinician-collected tests are not possible. These HP perceptions are consistent with self-collection implementation in Argentina: The strategy is used in the public health sector of several provinces for women with low access to screening services, and has been shown to increase coverage among socially vulnerable women [39].

One main finding of the study is that HPs decide their clinical practice based on several influences, including what they have learned during their training as gynecologists and local recommendations, especially those from their local PMAs. This finding is consistent with a study conducted in the United States to analyze patterns in the use of guidelines for breast and CC screening. The study found that gynecologists were mainly influenced in their practice by recommendations developed by their professional societies [11]. In Argentina, this influence of PMAs occurs in a regulatory context where recommendations by national health authorities do not entail punitive actions if not followed, and adherence to programmatic guidelines is relatively low [9]. This situation is shared by several low-middle-income countries [40, 41]. Therefore, a main implication of the study is that to accelerate the implementation of HPV-testing WHO should target and work with local PMAs, for example involving them in local adaptations of WHO guidelines. These professional associations have different roles based on which country they are located in and the healthcare system in which they operate: education and training including continuing medical education and certifications, licensing, regulation, ethical issues, setting standards including clinical guidelines, and representing doctors’ interests [42]. Therefore, they have a considerable influence on the professional life of HPs shaping their clinical practice and values regarding what interventions should or should not be implemented given the local context. Understanding the process through which local PMAs elaborate their recommendations would provide valuable evidence on how to approach and work with them to accelerate the incorporation of HPV-testing in the modality that is more adequate for each country’s socioeconomic and health resources situation. For example, targeting PMAs would be key in settings where implementation of the screen-and-treat approach should be the main strategy, as their non-acceptance will probably influence HP adoption more than its recommendations by national health authorities.

## Limitations

One limitation of the research is that interviewed health professionals worked in AMBA, which will somewhat limit the transferability of the results. Therefore, further research will be needed to evaluate the perspectives of health professionals from other provinces.

Also, our study did not interview other key stakeholders involved in the implementation of the 2021 WHO guidelines. However, it allowed us to identify PMAs as a main influence in health professionals’ decisions regarding using CC guidelines. Thus, the study results have provided key information for the design of the second phase of the Guides Project, which will evaluate PMAs’ perspectives on CC guidelines. Phase 2 of the Guides project is planned to begin in June 2024, funded by the WHO.

## Conclusion

Our study showed that HPs had little knowledge regarding WHO screening guidelines, although they highly valued this institution as a scientific developer of recommendations. They had a positive assessment of HPV-testing as primary screening every 5 years but were opposed to increased intervals and the screen-and-treat approach. Dissemination of WHO guidelines among HPs should be widely carried out, including its elaboration process and the evidence supporting them, especially in settings that could benefit from a screen-and-treat approach. The results allowed for identifying local PMAs as key influencers of HPs’ decisions regarding screening/diagnosis/treatment practices. Therefore, it is essential to generate evidence about how PMAs produce their recommendations and identify areas of partnership and collaboration to implement WHO guidelines. These results have important implications for countries implementing or planning to implement HPV -testing, as using WHO evidence-based recommendations will allow a more focused allocation of efforts and resources to populations facing barriers to screening, resulting in increased health equity and faster elimination of CC.

### Electronic supplementary material

Below is the link to the electronic supplementary material.

Supplementary Material 1: Consolidated criteria for reporting qualitative studies (COREQ): 32-item checklist; This reports the checklist used to describe methodological issues in data collection and analysis

## Data Availability

No datasets were generated or analysed during the current study.

## Abbreviations

AMBA: Metropolitan Area of Buenos Aires
CABA: Autonomous City of Buenos Aires
CC: Cervical cancer
CFIR: Consolidated Framework for Implementation Research
GBA: Great Buenos Aires
HPs: Health providers
HPV: Human papillomavirus
HPV+: Human papillomavirus positive
IARC/WHO: International Agency for Research on Cancer/World Health Organization
PAHO: Pan American Health Organization
PMAs: Professional medical associations
SITAM: National screening information system
U.S.: United States
UNICEF: United Nations International Children’s Emergency Fund
WHO: World Health Organization
