# Supplementary material for: Implementation of WHO guidelines for cervical cancer screening, diagnosis and treatment: knowledge and perceptions of health providers from Argentina
Source: BMC Cancer. 2024 Aug 12;24:996. doi: 10.1186/s12885-024-12650-7 (PMC11321033; doi:10.1186/s12885-024-12650-7)
Supplement: Supplementary file 1 — Supplementary Material 1: Consolidated criteria for reporting qualitative studies (COREQ): 32-item checklist; This reports the checklist used to describe methodological issues in data collection and analysis [file 12885_2024_12650_MOESM1_ESM.docx]

**Table 1 Consolidated criteria for reporting qualitative studies (COREQ): 32-item checklist**

No Item Guide questions/description

**Domain 1: Research team and reflexivity**

Personal Characteristics

1. Interviewer/facilitator: Which author/s conducted the interview or focus group?

Cecilia Straw (Author)

2. Credentials: What were the researcher’s credentials?

PhD

3. Occupation: What was their occupation at the time of the study?

Independent Researcher

4. Gender: Was the researcher male or female?

Female

5. Experience and training: What experience or training did the researcher have?

Researcher in social sciences applied to the field of health.

Relationship with participants

6. Relationship established: Was a relationship established prior to study commencement?

No.

7. Participant knowledge of the interviewer: What did the participants know about the researcher?

The participants were informed that the researcher was doing interviews as part of the Guides study.

8. Interviewer characteristics: What characteristics were reported about the interviewer/facilitator? e.g. Bias, assumptions, reasons and interests in the research topic

We reported that the interviewer was a researcher trained in qualitative research.

**Domain 2: study design**

Theoretical framework

9. Methodological orientation and Theory: What methodological orientation was stated to underpin the study?

Thematic analysis.

Participant selection

10. Sampling How were participants selected?

The sample was purposive.

11. Method of approach: How were participants approached?

The participants were approached through mail or WhatsApp to invite them to participate in a virtual interview, which was carried out through a virtual platform.

12. Sample size: How many participants were in the study?

15 participants: 15 health providers.

13. Non-participation: How many people refused to participate or dropped out? Reasons?

No person refused to participate or dropped out the interview.

Setting

14. Setting of data collection: Where was the data collected?

The data was collected through a virtual platform and most of the interviewees were at their workplaces.

15. Presence of non-participants: Was anyone else present besides the participants and researchers?

No other person was present during the interviews besides the respondent and the researcher.

16. Description of sample: What are the important characteristics of the sample?

The most important characteristic of the sample was that they were health providers who performed the screening, diagnosis, or treatment of cervical cancer.

Data collection

17. Interview guide:

Were questions, prompts, guides provided by the authors? Was it pilot tested?

The interviews were conducted using a guideline with questions prepared considering the relevant dimensions and constructs of the CFIR. To streamline the interview, participants were shown two cards that graphed the elaboration process of the evidence supporting the cervical cancer guidelines, and summary recommendations for screening and treatment approaches. The guideline was not pilot tested.

18. Repeat interviews: Were repeat interviews carried out? If yes, how many?

No interview was repeated.

19. Audio/visual recording: Did the research use audio or visual recording to collect the data?

Yes, the research used audio recording.

20. Field notes: Were field notes made during and/or after the interview or focus group?

The research did not make fields notes during the interviews.

21. Duration: What was the duration of the interviews or focus group?

The duration of the interviews was one hour on average.

22. Data saturation: Was data saturation discussed?

Yes.

23. Transcripts returned: Were transcripts returned to participants for comment and/or correction?

No.

**Domain 3: analysis and findings**

Data analysis

24. Number of data coders: How many data coders coded the data?

Transcripts were coded independently by two researchers’ (authors CS and VSA). Them both researchers met to review themes to identify consistencies and resolve the inconsistencies with the other members of the team (authors MP and SA). Several group meetings were held to discuss results and prepare the final manuscript.

25. Description of the coding tree: Did authors provide a description of the coding tree?

Yes. Table 1 of the manuscript presents the domains, constructs and theoretical dimensions that guided the coding.

26. Derivation of themes: Were themes identified in advance or derived from the data?

The themes were identified in advance in the guidelines prepared considering the dimensions and the relevant theoretical constructs.

27. Software: What software, if applicable, was used to manage the data?

The software used was ATLAS.ti (version 7.5.4; ATLAS.ti Scientific Software Development GmbH, Berlin).

28. Participant checking: Did participants provide feedback on the findings? No.

Reporting

29. Quotations presented: Were participant quotations presented to illustrate the themes / findings? Was each quotation identified?

Yes, quotes from the participants are presented to illustrate the themes. The citations identify the number of the respondent and the location where the health provider workplace (e.g., Professional 2, CABA; Professional 10, GBA).

30. Data and findings consistent: Was there consistency between the data presented and the findings?

Yes.

31. Clarity of major themes: Were major themes clearly presented in the findings?

Yes.

32. Clarity of minor themes: Is there a description of diverse cases or discussion of minor themes?

Yes.
